# Identification of Fangjihuangqi Decoction as a late-stage autophagy inhibitor with an adjuvant anti-tumor effect against non-small cell lung cancer

**DOI:** 10.1186/s13020-023-00770-4

**Published:** 2023-06-07

**Authors:** Qiugu Chen, Yuan Liao, Yujiao Liu, Yue Song, Junbo Jiang, Zhen zhang, Anqi Li, Mengyi zheng, Xiaoyi Chen, Tingxiu Zhao, Jiangyong Gu, Yuhui Tan, Xiaoyi Liu, Yanjun Jiang, Kun Wang, Hua Yi, Jianyong Xiao, Shan Hu

**Affiliations:** 1 Research Center of Integrative Medicine, School of Basic Medical Sciences, Guangzhou University of Chinese Medicine, Guangzhou, 510006 China; 2 Department of Pathology and Pathophysiology, Guangzhou University of Chinese Medicine, Guangzhou, 510006 China; 3 Department of Medical Biotechnology, Guangzhou University of Chinese Medicine, Guangzhou, 510006 China; 4 The Second Clinical College, Guangzhou University of Chinese Medicine, Guangzhou, 510006 China; 5 Department of Anaesthesia and Intensive Care, The Chinese University of Hong Kong, Hong Kong, 999077 SAR China

**Keywords:** Fangjihuangqi decoction, Autophagy inhibition, Sensitization, Cisplatin, Paclitaxel

## Abstract

**Background:**

Clinically, although chemotherapy is one of the most commonly used methods of treating tumors, chemotherapeutic drugs can induce autophagic flux and increase tumor cell resistance, leading to drug tolerance. Therefore, theoretically, inhibiting autophagy may improve the efficacy of chemotherapy. The discovery of autophagy regulators and their potential application as adjuvant anti-cancer drugs is of substantial importance. In this study, we clarified that Fangjihuangqi Decoction (FJHQ, traditional Chinese medicine) is an autophagy inhibitor, which can synergistically enhance the effect of cisplatin and paclitaxel on non-small cell lung cancer (NSCLC) cells.

**Methods:**

We observed the changes of autophagy level in NSCLC cells under the effect of FJHQ, and verified the level of the autophagy marker protein and cathepsin. Apoptosis was detected after the combination of FJHQ with cisplatin or paclitaxel, and NAC (ROS scavenger) was further used to verify the activation of ROS-MAPK pathway by FJHQ.

**Results:**

We observed that FJHQ induced autophagosomes in NSCLC cells and increased the levels of P62 and LC3-II protein expression in a concentration- and time-gradient-dependent manner, indicating that autophagic flux was inhibited. Co-localization experiments further showed that while FJHQ did not inhibit autophagosome and lysosome fusion, it affected the maturation of cathepsin and thus inhibited the autophagic pathway. Finally, we found that the combination of FJHQ with cisplatin or paclitaxel increased the apoptosis rate of NSCLC cells, due to increased ROS accumulation and further activation of the ROS-MAPK pathway. This synergistic effect could be reversed by NAC.

**Conclusion:**

Collectively, these results demonstrate that FJHQ is a novel late-stage autophagy inhibitor that can amplify the anti-tumor effect of cisplatin and paclitaxel against NSCLC cells.

**Supplementary Information:**

The online version contains supplementary material available at 10.1186/s13020-023-00770-4.

## Introduction

NSCLC accounts for a high proportion of all lung cancer cases, and is associated with a high fatality rate [1]. Most patients are no longer able to undergo surgical resection when diagnosed, and chemotherapy remains the most common treatment for NSCLC [2]. Although first-line clinical chemotherapy drugs can prolong the survival of NSCLC Patients to a certain extent, long-term drug use is prone to drug resistance with a greatly reduced chemotherapy effect. Moreover, a high dosage of these drugs will cause serious damage to normal tissues [3, 4]. Therefore, there remains an urgent need to identify effective and safe drugs for the prevention and treatment of NSCLC.

Cisplatin and paclitaxel are commonly used as first-line chemotherapeutic agents in clinical practice, and their activity induces increased levels of autophagy in NSCLC cells. Although autophagy is an important life process involved in the maintenance of normal cell homeostasis and can actively remove excess, damaged, senescent proteins and organelles to achieve self-renewal [5], it is a double-edged sword for tumors. During the early stage of cancerogenesis, autophagy has significant potential for cellular protection and tumor inhibition [6]. Nevertheless, if tumorigenesis has been initiated, autophagy can further sustain tumor development [7]. Moreover, many malignant tumors require autophagy to promote oncogenic processes (e.g. autophagy supports ErBB2-driven tumorigenesis in breast cancer) [8]. Autophagy also protects tumor cells attacked by chemotherapy drugs or radiotherapy radiation to prevent apoptosis [9]. Therefore, it is reasonable to hypothesize that increased chemotherapy sensitivity of tumor cells may be achieved by inhibiting autophagy.

In general, according to the different stages of autophagy inhibition, available autophagy inhibitors can be divided into two categories: (1) those that inhibit autophagy induction during the early stage of autophagy, such as the frequently-used 3-methyladenine (3-MA) and wortmannin [10, 11]; and (2) those which block the late stages of autophagy. Bafilomycin A1 (BafA1) is an inhibitor of vesicle-type ATPase, which can interfere with the fusion of autophagy vesicles and lysosomes, as well as inhibit lysosomal acidification, affecting cathepsin maturation [12]. Unfortunately, due to their high toxicity, the use of autophagy inhibitors is limited in clinical applications. For example, HCQ (classic clinical autophagy inhibitor) has been reported to cause retinal toxicity [13]. Therefore, the development of more effective autophagy inhibitors is of great clinical significance.

With their highly diverse biological activities and functions, Chinese botanical drugs play a crucial role in the development of drugs to treat various diseases [14–16]. Indeed, some newly discovered drugs with anti-tumor activity are known to have originated from traditional Chinese medicine. For example, Mufangji Decoction can inhibit tumor growth through regulating Akt/mTOR-mediated autophagy in NSCLC [17]. However, the pool of traditional Chinese medicine is very large, and there are many effective anti-tumor drugs to be explored. Fangjihuangqi Decoction (FJHQ) is a traditional Chinese classical medicine prescription with anti-inflammatory, antipyretic function that supports immune function, which is consistent with the discussion of the etiology and pathogenesis of lung cancer in TCM [18]. Additionally, it has been reported that FJHQ elicits a therapeutic effect in the treatment of TNBC by upregulating E-cadherin while downregulating EMT markers [19]. However, whether FJHQ is effective against NSCLC and the associated mechanism remains unclear.

In our study, the adjuvant anti-tumor effect of FJHQ as a new type late-stage autophagy inhibitor against NSCLC was explored. It was found that FJHQ can block autophagy by inhibiting the maturation of cathepsin in lysosomes and induce the accumulation of ROS in tumor cells. Increased ROS further activates the ROS-MAPK pathway and induces apoptosis. Thus, FJHQ enhances the anticancer effects mediated by cisplatin and paclitaxel.

## Materials and methods

### Preparation of FJHQ

FJHQ is composed of Stephania tetrandra S.Moore (Menispermaceae, fang ji), Astragalus mongholicus Bunge (Fabaceae, huang qi), Atractylodes macrocephala Koidz (Asteraceae, bai zhu), Glycyrrhiza glabra L (Fabaceae, gan cao), Zingiber officinale Roscoe (Zingiberaceae, fresh ginger) and Ziziphus jujube Mill (Rhamnaceae, da zao) with a ratio of 4:5:3:2:3:1. The medicine was purchased from Guangzhou Kangmei Wisdom Pharmacy and was validated by Prof. Zhongxiang Zhao at Guangzhou University of Chinese Medicine.

432 g of FJHQ was soaked in 10 times the volume of ddH_2_O (4320 ml) for 1 h. After decoction and filtration, the filtrate was concentrated to 432 ml with a concentration of 1 g/mL.54 ml of drug juice was freeze-dried to powder (− 40 ℃, 200MT, 24 h) under a vacuum and was stored at − 20°.When using, dissolved it with DMEM (C11995500BT, Gibco). The remaining was converted to the equivalent dose of mice according to the adult dosage (770 mg/kg), diluted with ultrapure water (702 mg/mL), and gavage 200 μL daily.

The quality control of FJHQ was conducted by UPLC/Q-TOF–MS/MS, using Shimadzu CBM20Alite Controller, equipped with Waters Acquity UPLC BEH C_18_ column (100 × 2.1 mm, 1.7 µM). The mobile phase consisted of 0.1% formic acid-acetonitrile (A), 0.1% formic acid—water (B). Linear gradient elution procedure: 0–0.5 min, 10% A; 0.5–7 min, A 10–27%; 7–10 min, 27–82% A; 10–16 min, 82% A; 16–18 min, 82–100% A; 18–22 min. 100%. The flow rate was 0.4 mL/min, and the injection volume was 3 µL.The column temperature was 30 °C.

Metabolomic analysis was performed on an AB SCIEX Triple TOF^™^ 5600^+^ (Foster City, CA). The raw data was obtained by using Analyst^®^TF 1.7 software (AB Sciex, Foster City, CA). The mass scan range of TOF–MS was 100–1500 Da and 50–1250 Da for TOF–MS/MS. Its ion accumulation time was 250.0 ms. 550 °C was adjusted for a turbo spray temperature. Curtain gas was 35 psi, heater gas was 55 psi, nebulizer gas was 55 psi and the ion spray voltage was 4500 V. The IDA (information dependent acquisition) standard is set for 8 more intense peaks, with intensity greater than 100  cps.

### Chemicals

Bafilomycin A1 (S1413), Cisplatin (S1166) and N-acetyl-L-cysteine (NAC, S1623) were purchased from Selleck Chemical (Houston, TX, USA); Paclitaxel (A0177) was purchased from Chengdu Must Bio-Technology (Chengdu, China). With the exception of cisplatin, which was dissolved in dimethylformamide, all other compounds were dissolved in dimethyl sulfoxide (DMSO) and stored at a temperature of − 80 °C.

### Cell culture

NCI-H1299 (ATCC^®^CRL-5803^™^) and NCI-H1975 (ATCC^®^ CRL-5908^™^) are purchased from American Type Culture Collection (ATCC), The cells were cultured in DMEM containing 10% FBS (FSP500, Excell-bio) and 1% penicillin–streptomycin solution (P1400, Solarbio) at 37 °C in 5% CO_2_ atmosphere.

### Cell viability assay (CCK8)

Cells were seeded in 96-well plates at a density of 7 × 10^3^ cells/well and cultured for 24 h. The combined culture group was pretreated with FJHQ (800 μg/mL) for 4 h and then cisplatin or Paclitaxel were added in a concentration gradient for 24 h. Finally, 10 μL of Cell Counting kit-8 (CCK8, 40203ES80, Shanghai YiSheng) reagent was added to each well, and the Cell viability was measured by optical density at 450 nm.

### Western blot analysis

Total protein was extracted by lysis of cells with SDS-PAGE Sample Loading (P0015, Beyotime), and 20 μl/ lane of protein extract was separated on SDS–polyacrylamide gels and then transferred onto PVDF membranes (0.22 µm pore, Roche). After that, the membranes were blocked with TBST buffer (20 mM Tris, 137 mM NaCl, 0.1% Tween-20, pH8.0) containing 5% nonfat milk for 3 h, then coated with primary antibody and placed at 4 °C overnight and after that were incubated at room temperature for 1 h. Protein bands were visualized with Immobilon Western chemiluminescence HRP substrate.

Primary antibody information: β-actin(3700, CST), P62(88588, CST), LC3-A/B(12741, CST), cathepsin B(31718S, CST), cathepsin D(2284, CST), cathepsin D(AF1014, R&D), cathepsin L(AF952, R&D), PARP(5625, CST), JNK(9258, CST), P-JNK(4668, CST), P-P38(4511, CST), P38(8690, CST), Caspase 3 (GTX110543,GTX). All were diluted 1:1500.

Secondary antibody information: anti-mouse antibody (AS003, Abclonal) and anti-rabbit antibody (AS014, Abclonal), both were diluted 1:5000.

### Autophagosome and lysosome colocalization

NCI-H1299 or A549 transfected with GFP-LC3 was seeded in confocal dish (5 × 10^4^ cells/well). After 24 h, Lyso Brite^™^ Red (22645, AAT Bioquest) was added and then placed the plate in the cell incubator to staining. After 20 min, the plate was washed three times with PBS. Using a laser con-focal scanning microscope (X63 objective lens, LSM 800; Carl Zeiss, Jena, Germany) to capture images.

### Annexin-V/PI apoptosis assay

The cells were seeded in 6-well plates (1.5 × 10^5^ cells/well) and cultured overnight. Then after treating the cells in different groups for 24 h, the cells were trypsinized、collected and washed twice with PBS. Annexin-V (3 μL) and PI (5 μL) (Annexin V FITC Apop Dtec Kit I 100Tst, 556547, BD) were added and the cells were to infiltrate in the dark for 20 min and 5 min. Finally, apoptotic cells were counted by BD Accuri C6 flow cytometry. FITC + /PI– and FITC + /PI + groups were considered to be apoptotic cells.

### Intracellular ROS detection

After the cells were seeded and treated with drugs for 24 h, the cells were harvested and resuspended twice with basic DMEM. Labeled cells with 10 μM H2DCFDA (D399, Thermo Fisher) at 37 °C for 20 min, and then the intracellular ROS levels were determined by flow cytometry.

### Human lung cancer xenografting

This experiment was approved by the Animal Ethics Committee of Guangzhou University of Chinese Medicine (Approval No. 20220516003). Male BALB/C nude mice aged 6–7 weeks, weighing 17–20 g, were purchased by Guangdong Medical Laboratory Animal Center and raised in SPF animal laboratory. After 1 week of feeding with sufficient sterile water and food, NCI-H1299 (4 × 10^6^/200 μL) was injected into the right posterior back. When the tumor reached 0.1 mm3, the mice were randomly divided into 4 groups (n = 8 per group) and treated as follows: Pure water + saline solution, FJHQ (7.020 mg/g) + saline solution, pure water + cisplatin (0.75 mg/kg), FJHQ + cisplatin (Before cisplatin, FJHQ was administered for three days). During the experiment, the tumor volume was measured every 2 days, and the mice were sacrificed for dissection and tumors were weighed after 18 days of drug combination.

### Statistical analysis

All the conclusions are based on three independent experiments. Excel and GraphPad Prism were used to analyze the original data and plot the results. The data met the assumption of normal distribution. Multiple groups were calculated by one-way analysis of variance, the mean ± S.D. was expressed. Student’s t test was used for statistical analysis when the variance between two groups was similar. *P < 0.05, **P < 0.01, and ***P < 0.001 all indicated statistically significant differences. The synergistic inhibitory effect of drug combination was analyzed by Q value, Q = Eab/(Ea + Eb − Ea × Eb), Q value > 1.15 indicated synergistic relationship. The value of Q was between 0.85 and 1.15, indicating additive relationship. Q value < 0.85 indicates an antagonistic relationship.

## Results

### Analysis of main Fangjihuangqi decoction components by HPLC

**Fig. 1:**
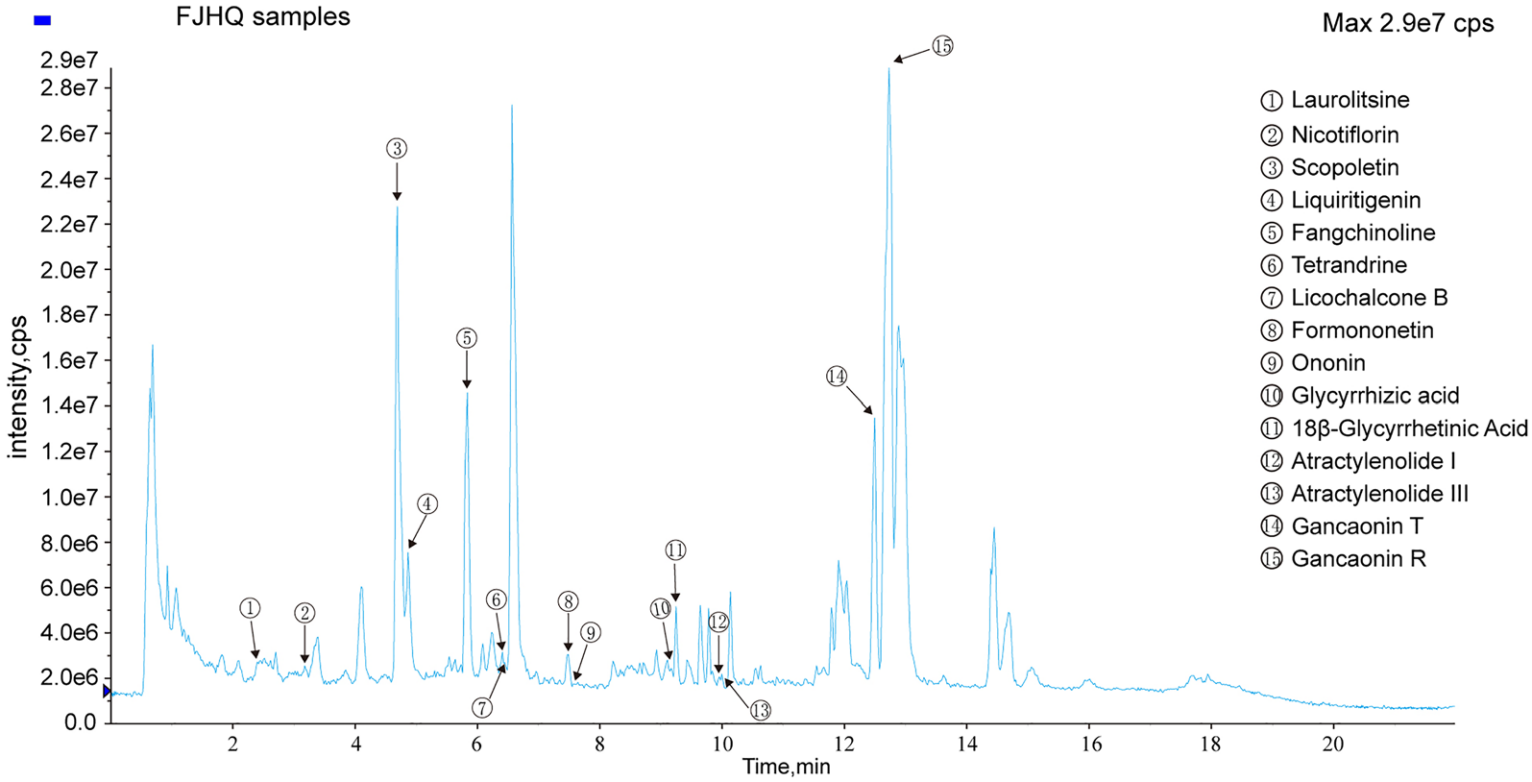
Total ion chromatograms chromatograms of FJHQ by UPLC-Q-TOF-MS/MS and its representative compounds

To determine the main components in Fangjihuangqi decoction, we used UPLC/Q-TOF-MS/MS in our analysis. The method was simple and selective. Moreover, total ion chromatograms the FJHQ extract is shown in Fig. 1 and the detailed information of each component is presented in Additional file 1: Fig. S1.

### FJHQ inhibits the late stage of autophagic flux in NSCLC

**Fig. 2:**
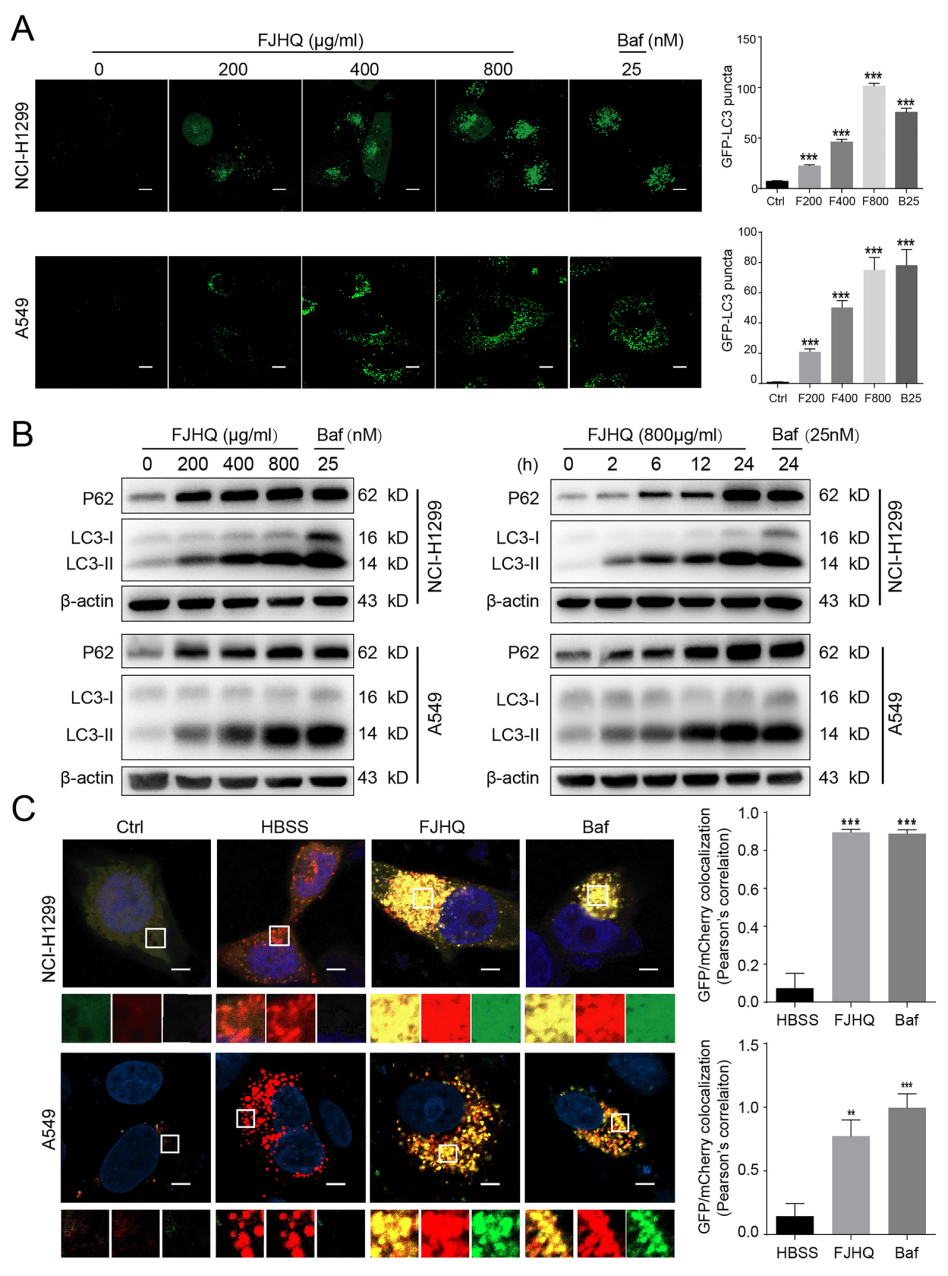
FJHQ inhibits the late-stage autophagic flux in NSCLC cells. **A** FJHQ and BafA1 induce a large accumulation of LC3-GFP puncta. The bar graph shows the number of LC3-positive spots per cell (n = 20). **B** FJHQ results in increased P62 and LC3-I to LC3-II conversion in the form of a time and concentration gradient. **C** HBSS treatment triggers mCherry puncta accumulation but few GFP-LC3 puncta. After FJHQ and Bafilomycin A1 treatment, the colocalization of red and green fluorescence was significantly higher compared to HBSS. Twenty cells were randomly selected from each group, the number of red-only, yellow or total puncta was counted. ***P < 0.001, ANOVA with multiple comparisons. Scale bar: 5 μm. *FJHQ* Fangjihuagnqi Decoction, *BafA1* Bafilomycin A1

LC3 is a marker of autophagosome formation. During the process of autophagy, cytoplasmic LC3 (lC3-I) will be transformed into the membrane type (LC3-II), LC3-II is attached to the inner membrane of the autophagosome until it is eventually degraded in the autophagolysosome [20]. Initially, to examine the effect of FJHQ on autophagy, we detected the number of LC3-positive puncta on GFP-LC3-transfected NCI-H1299 and GFP-LC3-transfected A549, and found that it increased with the concentration gradient of FJHQ. This effect was found to be similar to bafilomycin A1 treatment (Fig. 2A), suggesting that FJHQ may regulate autophagy. We next detected the changes of LC3 conversion. The level of lc3-II protein expression was upregulated in a time- and dose-dependent manner both in NCI-H1299 and A549 (Fig. 2B), indicating an increased number of autophagosomes in NSCLC following FJHQ treatment.

The increased level of LC3-II and number of GFP-LC3 puncta can be related to either increased autophagosome formation or impaired autophagosome degradation. To distinguish between these two possibilities, we measured the level of P62, a protein that binds to ubiquitinated proteins and forms a complex with the LC3-II protein, and are eventually degraded together in the autolysosome [21]. Therefore, elevated levels of P62 commonly reflect a blocked autophagic flux. The P62 protein level was elevated by FJHQ in a concentration- and time-dependent manner (Fig. 2B). Therefore, the simultaneous upregulation of LC3-II and P62 indicates that the increase in autophagosomes was due to its decreased degradation, suggesting that FJHQ might block autophagic flux during the later stage.

To further confirm that FJHQ affects the degradation stage of autophagy, we transfected NCI-H1299 and A549 with mCherry-GFP-LC3B tandem structure, a widely accepted method used to visualize autophagic flux. Essentially, if autophagy proceeds normally, starvation induction by HBSS appears as a greater number of red dots as green GFP is quenched by the acidic environment of the lysosome. In contrast, interference with autophagosome-lysosome fusion or disruption of lysosome function causes most of the spots to appear yellow because they fluoresce as both red and green. As expected, FJHQ significantly increased the number of yellow puncta in both types of cells, which was similar to the effects of Bafilomycin A1, indicating an impaired autophagic flux. Inversely, multiple red puncta with few green puncta were observed in the HBSS-incubated cells, indicating an efficient autophagy response (Fig. 2C). Collectively, these data confirmed that FJHQ is a late-stage autophagy inhibitor.

### FJHQ interrupts autophagic flux by inhibiting cathepsin maturation instead of blocking autophagosome-lysosome fusion

**Fig. 3:**
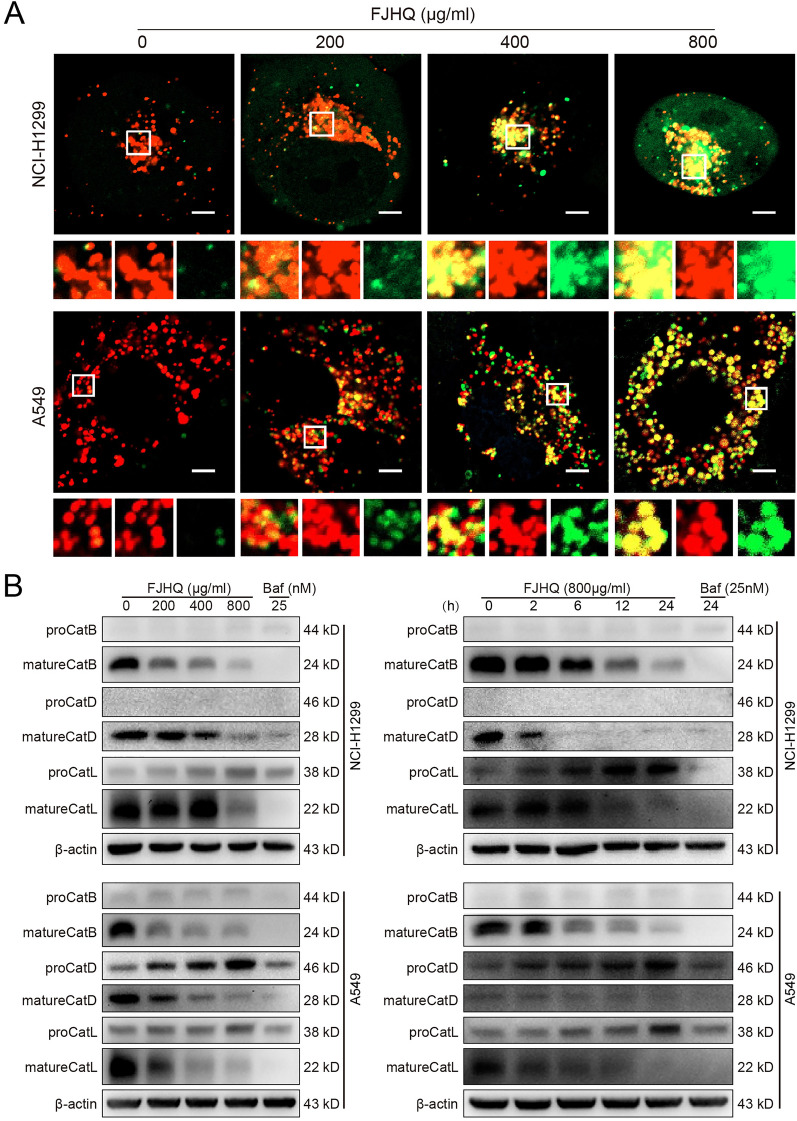
FJHQ inhibits cathepsin maturity. **A** NCI-H1299 and A549 stably expressing GFP-LC3 were stained with Lyso Brite^™^ Red (lysosomal labeling), and the colocalization of fluorescent spots (yellow) was significantly increased after FJHQ treatment as the concentration gradient. Scale bar: 5 μm. **B** FJHQ affects Cathepsin maturation. Fangjihuangqi Decoction: FJHQ; bafilomycin A1: BafA1

Since FJHQ blocks the progression of late autophagic flow, it must be determined whether FJHQ inhibits autophagy by blocking autophagosome-lysosome fusion or impairing autolysosome degradation. To address whether FJHQ affects the fusion of autophagosomes with lysosomes, we next assessed the colocalization of GFP-LC3 and Lyso Brite^™^ Red (a fluorescent marker used to label and track acidic organelles in live cells, such as autolysosomes) in GFP-LC3-transfected NCI-H1299 and GFP-LC3-transfected A549. We found that most GFP-LC3 puncta were colocalized with Lyso Brite^™^ Red after treatment with FJHQ as the concentration increased and appeared as yellow spots (Fig. 3A). This finding indicated the colocalization of lysosomes and autophagosomes. Together, these observations confirmed that FJHQ did not suppress autophagosome-lysosome fusion.

Lysosomal enzymes play an important role in autophagic degradation. The failure of autophagy substrates to be degraded may be related to decreased cathepsin maturity. Next, we examined whether FJHQ treatment affects lysosomal enzymes expression, which produced from precursor forms (pro-cathepsins), into the mature-cathepsins [22]. As shown in Fig. 3B, the levels of mature-CatB 、mature-CatD and mature-CatL at protein expression were reduced both in NCI-H1299 and A549 in a dose-and time-dependent manner, collectively demonstrating that FJHQ disturbs autophagy by inhibiting lysosomal cathepsin maturity in NSCLC cells.

### FJHQ enhanced the anti-cancer effects of cisplatin and paclitaxel

Cisplatin and paclitaxel represent first-line chemotherapeutic agents used for the clinical treatment of NSCLC. Its mechanism of action is to cause mitochondrial damage in tumor cells to release ROS and further activate apoptotic pathways [23, 24]. In fact, tumor cells can eliminate damaged mitochondria through the up-regulation of autophagy activity to reduce apoptotic factors and thereby avoid killing [25, 26], resulting in drug resistance of tumor cells and reducing drug efficacy. Based on this resistance phenomenon, it appears that autophagy induced by cisplatin and paclitaxel is cytoprotective in nature. Therefore, autophagy inhibition is a strategy to alleviate drug resistance in tumor therapy.

**Fig. 4:**
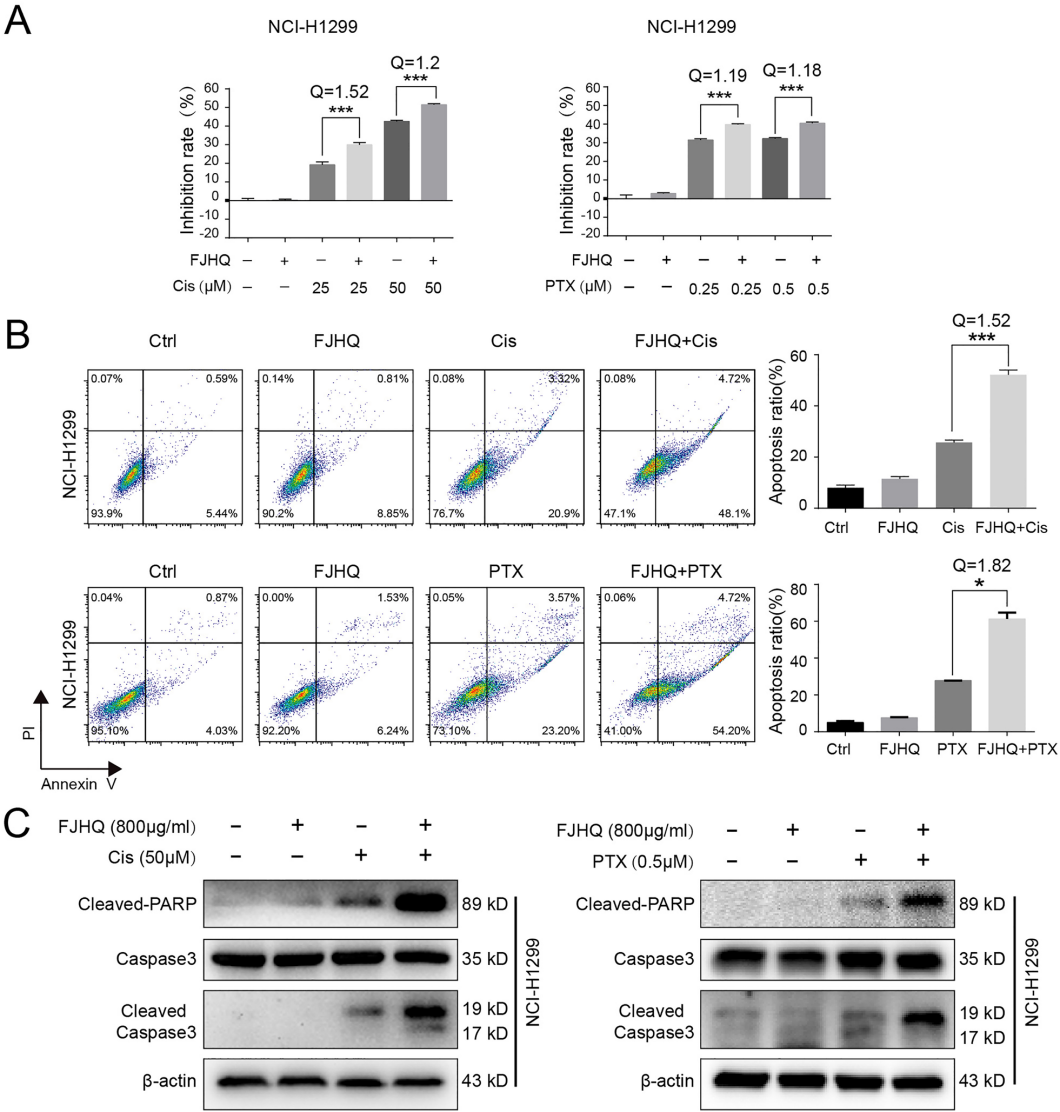
FJHQ enhances cisplatin (cis)– and Paclitaxel-mediated anticancer effects. **A** FJHQ (800 μg/mL) showed no or little cytotoxic effect on NCI-H1299. Pretreatment with FJHQ (800 μg/mL) enhanced cis-mediated inhibition at all tested concentrations (25 and 50 μM) and Paclitaxel -mediated inhibition at all concentrations tested (0.25 and 0.5 μM). (^*^P < 0.05; ^**^P < 0.01; ^***^P < 0.001, multiple comparison ANOVA; Q > 1.15, indicated a synergistic effect). **B** The apoptosis rate induced by FJHQ combined with cis was significantly higher than that induced by cisplatin or paclitaxel alone (^*^ *P* < 0.05; ^**^ *P* < 0.01; ^***^P < 0.001, multiple comparison ANOVA; Q > 1.15, indicated a synergistic effect). **C** Pre-treatment with FJHQ promotes cis- and paclitaxel-triggered Caspase-3 and PARP cleavage. *FJHQ* Fangjihuangqi Decoction, *Cis* Cisplatin, *PTX* Paclitaxel

Since our data indicate that FJHQ can inhibit autophagy, we next examined whether FJHQ can play the same synergistic effect in combination with chemotherapy drugs. The CCK8 assay revealed that FJHQ combined with different doses of cisplatin or paclitaxel significantly inhibited NCI-H1299 cells proliferation compared with each drug alone (Fig. 4A). Figure 4B shows that FJHQ effectively enhanced cisplatin- and paclitaxel-induced apoptosis, and the Q value (> 1.15) indicated that combination therapy had a synergistic inhibitory effect. More importantly, both cisplatin and paclitaxel increased the levels of cleaved Caspase-3 and cleaved PARP in NCI-H1299 cells, and these apoptotic markers were further increased when combined with FJHQ (Fig. 4C). Therefore, FJHQ may enhance the tumor killing effect of cisplatin and paclitaxel by inhibiting autophagy.

### FJHQ combined with cisplatin or paclitaxel can significantly promote mitochondrial ROS

Cisplatin- and paclitaxel-induced damaged mitochondria will lead to a temporary accumulation of intracellular reactive oxygen species (ROS), and high concentration of ROS play a key role in the induction of apoptosis [27, 28]. However, up-regulated autophagic flux increases the clearance of damaged mitochondria so that autophagy indirectly represents an important ROS scavenger. Therefore, autophagy inhibition is likely to lead to excessive ROS accumulation in cells treated with chemotherapy drugs, enhancing their killing effect on cancer cells. Therefore, we first examined whether cisplatin or paclitaxel combined with FJHQ increased ROS accumulation in NSCLC cells by inhibiting autophagy.

**Fig. 5:**
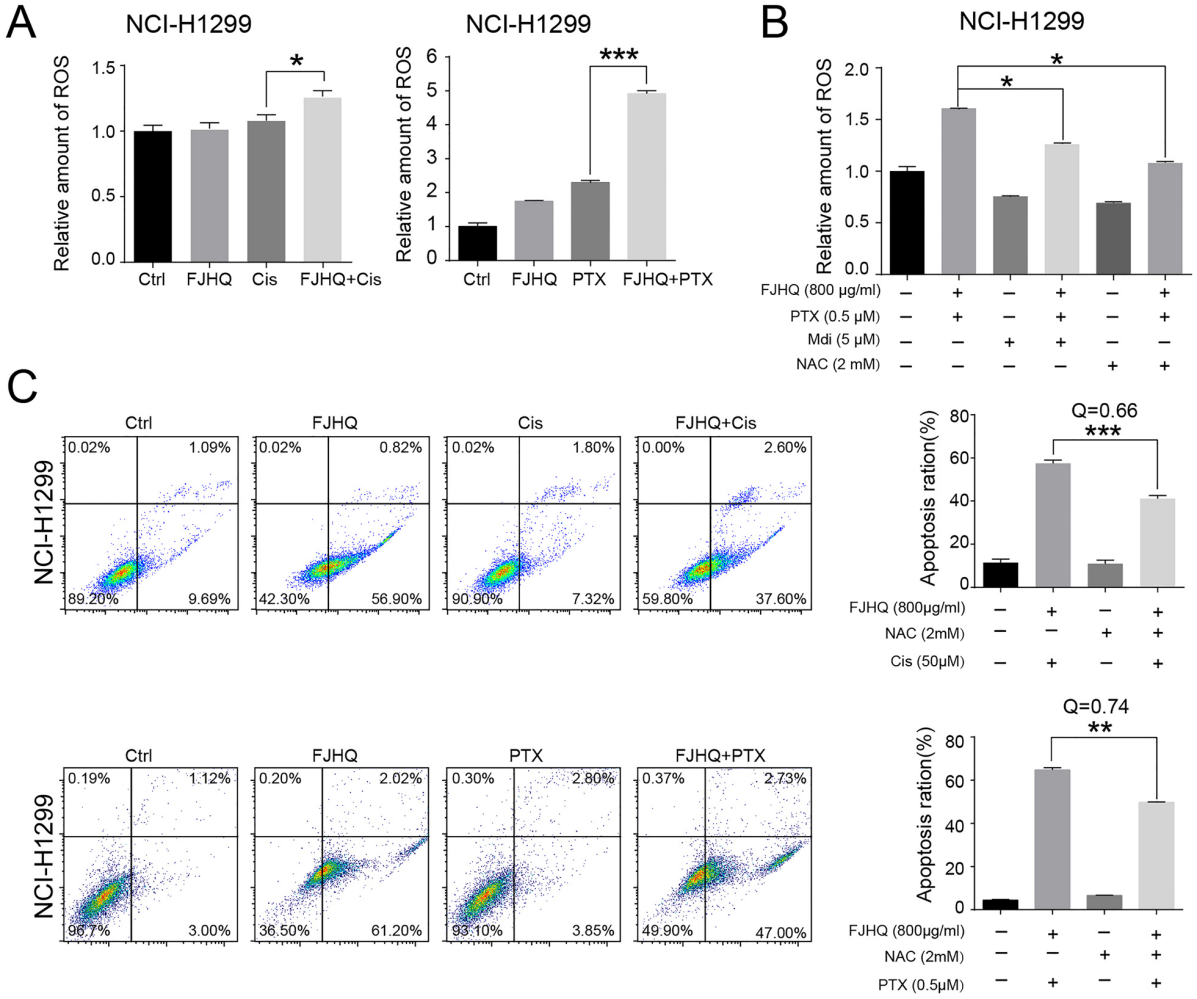
FJHQ combined with cisplatin and paclitaxel can significantly promote mitochondrial ROS. **A** Compared with cisplatin (50 μM) and paclitaxel (0.5 μM) treating alone, Pre-treatment with FJHQ (800 μg/ml) significantly increased the Cis-induced ROS production and Paclitaxel-induced ROS production in NCI-H1299. **B** Excessive accumulation of ROS accumulation arises from damaged mitochondria, and NAC can reduce ROS accumulation. **C** The apoptosis rate of FJHQ combined with cisplatin or paclitaxel could be reversed by NAC (^*^ *P* < 0.05; ^**^ *P* < 0.01; ^***^P < 0.001, multiple comparison ANOVA and Q < 0.85 indicated an antagonistic effect)

As we can see that ROS was higher in cisplatin or paclitaxel treating groups than in the non-drug group and more importantly, FJHQ in combination with cisplatin or paclitaxel significantly increased the ROS levels compared with those chemotherapy drugs treated alone in NCI-H1299 (Fig. 5A), suggesting that an inhibition of autophagy by FJHQ resulted in increased intracellular ROS. It is possible that the accumulation of damaged mitochondria resulting from the inhibition of autophagy by FJHQ contributes to this phenomenon. Next, we explored whether the increased level of ROS was derived from damaged mitochondria. As Fig. 5B illustrates, the mitochondrial division inhibitor Mdivi-1 (Mdi) was found to effectively reverse the mitochondrial fragmentation induced by the combination of FJHQ and paclitaxel, and to reduce ROS accumulation to a degree comparable to that achieved by the ROS scavenger NAC. These results are consistent with our earlier speculation, and provide further evidence to suggest that the inhibition of autophagy by FJHQ leads to the accumulation of damaged mitochondria and an increase in mitochondrial ROS levels.

As we have previously established, the combination of FJHQ with either cisplatin or paclitaxel results in a synergistic increase in ROS accumulation in NSCLC cells. It is reasonable to speculate that ROS may play a crucial role in mediating this synergistic effect. We further examined the level of apoptosis after adding NAC. As shown in Fig. 5C, in NCI-H1299 cells, FJHQ combined with cisplatin for 24 h significantly induced cell apoptosis (57.50 ± 0.36%, Annexin V + /PI–), whereas the addition of NAC significantly reduced the apoptosis rate (57.50 ± 0.36% vs. 41.20 ± 0.31%; *P* < 0.001). The same situation was also observed in FJHQ combined with paclitaxel group (64.83 ± 0.58% vs 49.80 ± 0.10%; *P* < 0.01), and Q < 0.85 indicated an antagonistic effect. Therefore, ROS elimination can reverse the inhibitory effect of FJHQ combined with chemotherapy in NCI-H1299 cells.

These data indicate that autophagy may prevent the apoptosis of cancer cells by eliminating ROS or damaged mitochondria. FJHQ can inhibit this protective mechanism due to its synergistic role, which promoting ROS release and increased the rate of apoptosis in NSCLC cells.

### Excessive accumulation of ROS further activates the ROS-MAPK pathway

**Fig. 6:**
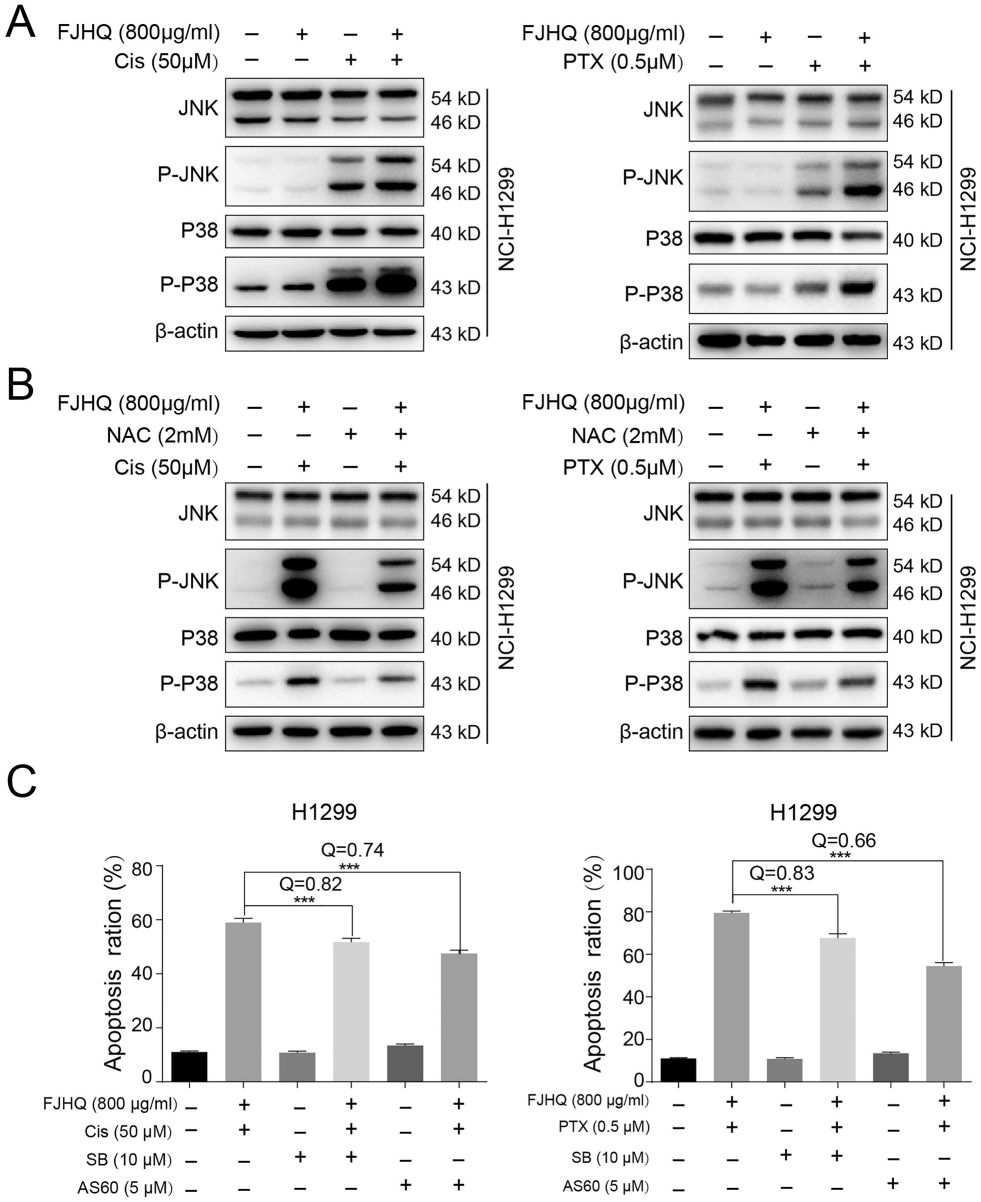
Excessive accumulation of ROS further activates the ROS-MAPK pathway. **A** The combination of cisplatin (50 μM) or paclitaxel (0.5 μM) with FJHQ (800 μg/mL) significantly increased the levels of JNK and P38 phosphorylation. **B** Increased P-JNK in the FJHQ + Cis or FJHQ + PTX groups was reversed by NAC. **C** AS601245 and SB203580 both reversed the synergistic effect of combined treatment of FJHQ with chemotherapy drugs (^***^P < 0.001, multiple comparison ANOVA and Q < 0.85 indicated an antagonistic effect). *FJHQ* Fangjihuangqi Decoction, *Cis* Cisplatin, *PTX* Paclitaxel

ROS-induced apoptosis usually occurs by activating the mitogen-activated protein kinase (MAPK) cascade, which primarily includes JNK and P38 kinase, both of which have similar functions in inflammation, apoptosis, and growth [29]. In this study, Western blotting further detected which kinases mediated related apoptosis, and the results showed that FJHQ combined with chemotherapy resulted in the up-regulation of phosphorylated JNK and P38 (Fig. 6A). The level of phosphorylation of these two proteins was significantly decreased after ROS was removed by NAC (Fig. 6B). To investigate whether the activation of JNK and p38 is responsible for the observed synergistic effect, we employed AS601245 (a JNK inhibitor) and SB203580 (a P38 inhibitor). The results demonstrated that both inhibitors effectively reversed the synergistic effect of the combined treatment with FJHQ and chemotherapy drugs, as shown in Fig. 6C. These findings suggest that the synergistic effect observed with the combined treatment of FJHQ and chemotherapy drugs may be mediated through the simultaneous activation of the JNK and P38 pathways.

### FJHQ enhances the inhibitory effect of cisplatin on transplanted tumors in vivo

**Fig. 7:**
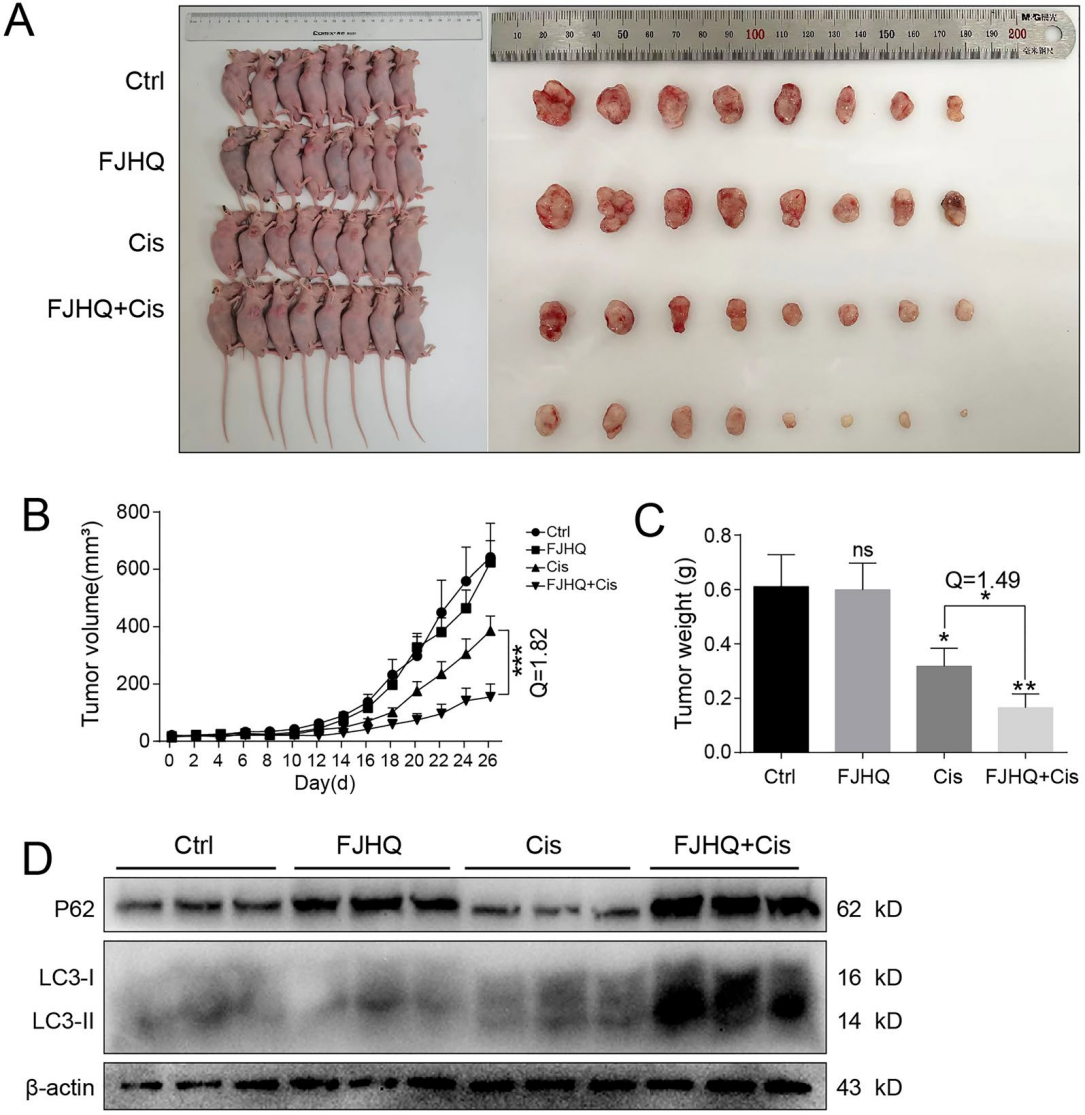
Co-treatment with FJHQ and cisplatin enhanced the inhibition of transplanted tumor in vivo*.* **A** Images showed NCI-H1299 injected-mice and subcutaneous tumors of all the four groups, FJHQ (7.020 mg/g) or Cis (0.75 mg/kg) or a combination of FJHQ and Cis. (FJHQ 7.020 mg/g with Cis 0.75 mg/kg). **B** Tumor volume was measured every 2 days and values were expressed as the mean ± S.D. (^***^P < 0.001; Q > 1.15 indicated a synergistic effect). **C** Bar chart depicting the weight of the tumors after dissection. (^*^P < 0.05; Q > 1.15 indicated a synergistic effect). **D** Compared with the no-drug group, P62 and LC3-II were increased in the FJHQ group, P62 was decreased in the Cis group, and LC3-II was increased. Compared with the FJHQ and Cis groups, P62 and LC3-II were significantly increased in the combined medication group. *FJHQ* Fangjihuangqi decoction, *Cis* cisplatin

To test whether FJHQ also functions as a synergist of cisplatin in vivo, BALB/C nude mice were used for in vivo tumor transplantation. NCI-H1299 was injected into male nude mice, and different therapeutic drugs were administered after the tumor had grown to 0.1 mm^3^. We observed that the tumors in the combined group were smaller than those in the other groups (Fig. 7A). Every 2 days, the tumor volume was measured, and the tumor growth rate of the cisplatin group was significantly slower than that of the no-drug group, and the combined administration of cisplatin further delayed the tumor growth rate and the Q > 1.15 (Fig. 7B). These findings indicated that FJHQ and cisplatin have a synergistic effect, whereas FJHQ itself had no effect on tumor growth. In addition, the tumors were weighed and the weight of the tumors in the combined drug group was lower than that in the cisplatin group (*P* < 0.05) (Fig. 7C). Subsequent western blot analysis was conducted to evaluate the expression of key autophagy markers in tumor tissues. The results revealed a simultaneous upregulation of both LC3-II and P62 in the combination group, which is consistent with the in vitro findings (Fig. 7D). Taken together, these results suggest that the combined treatment with FJHQ and cisplatin exerts a synergistic inhibitory effect on tumor growth in vivo, which is in agreement with the findings from the in vitro studies.

## Discussion

Targeting autophagy has emerged as a promising new strategy for the treatment of many cancers. Autophagy is a multistep process, each step of which can be inhibited. For example, ULK1, Pan-PI3K, and VPS34 inhibitors can be used in the early stage of autophagy [30–32]. Another approach of inhibiting autophagy is to target the later stages of the autophagy mechanism, including the inhibition of autophagosome-lysosome fusion and interference in lysosome function.

During the first part of our study, we identified FJHQ as an effective late-stage autophagy inhibitor, which disrupts the function of lysosomes and prevents autophagy substrates from being degraded smoothly. By combining several different approaches, we observed that FJHQ increases autophagy markers by inhibiting autophagic flux. The co-localization of GFP-LC3 (autophagosome marker) and Lyso Brite^™^ Red (lysosome marker) was observed following FJHQ treatment, suggesting that FJHQ did not affect the fusion of autophagosomes and lysosomes. Most recently-discovered late-stage autophagy inhibitors can impair lysosomal hydrolysis [33]. Cathepsin B, Cathepsin D, and Cathepsin L are among the most abundant lysosomal cysteine and aspartyl proteases, and are known to play a role in the degradation of autolysosomes [34–36], so we tested the maturity of the three cathepsins and found that FJHQ can significantly reduce the maturity of them. These results indicate that FJHQ depends on the damage of lysosomal function to interrupt autophagy. The functionality of FJHQ is somewhat similar to CQ. In contrast to previous studies, some recent studies have suggested that CQ does not actually reduce lysosome acidity, but rather causes a transient increase in lysosome pH and lysosomes retain their ability to degrade transport materials [37, 38]. This confusion is due to the fact that the commonly used LysoTracker Red is not a lysosome pH sensor, and the intensity of its fluorescence signal is independent of lysosomal pH [37]. The dynamics of this transient phase may vary between each cell type. In addition, CQ treatment led to lysosomal swelling [39]. Some late inhibitors (e.g., PIKfyve inhibitors) can lead to the enlargement of endolysosomes [40]. Interestingly, we observed that lysosomes labeled with acridine orange (AO) were significantly enlarged following FJHQ treatment compared with the untreated group. We suspected that FJHQ could also cause lysosomes to "expand". Moreover, we doubted that FJHQ treatment could greatly enhance lysosome membrane permeability (LMP), causing AO to leak from the lysosome into the cytoplasm, where it emits green fluorescence and allows for additional AO to enter the lysosome. Therefore, an interesting phenomenon of coexistence of red and green puncta was seen (data not shown). However, whether FJHQ affects lysosomal acidification remains to be further explored.

Another important observation of our study was that FJHQ combined with cisplatin or paclitaxel significantly enhanced the killing effect of NCI-H1299. Our findings indicate that the mechanism by which FJHQ inhibits autophagy to enhance the efficacy of chemotherapy drugs is not limited to NCI-H1299 cells, but also applies to EGFR mutant cell lines such as NCI-H1975. As shown in Additional file 2: Fig S2A, B, FJHQ treatment led to an increase in P62 and LC3-II levels, and a decrease in mature-catD, collectively indicating that FJHQ disrupts late-stage autophagy by inhibiting lysosomal cathepsin maturation in these cells. Moreover, Additional file 2: Fig S2C, D demonstrate that the combination of FJHQ and cisplatin can effectively inhibit the proliferation and increase the apoptosis rate of NCI-H1975 cells, which is also accompanied by an elevation in ROS levels (Additional file 2: Fig. S2E). Notably, the increased apoptosis rate could be reversed upon scavenging ROS with NAC, as shown in Additional file 2: Fig. S2F. This is based on the fact that treatment with these chemotherapy drugs increases the autophagic flux of NSCLC cells and vice versa [41]. Previous studies have shown that paclitaxel treatment cannot induce an increase in NCI-H1975 autophagic flux [42], which works for NCI-H1299. Therefore, paclitaxel does not increase the apoptosis rate of NCI-H1975 following combined use with FJHQ (data not shown). Cleaved-PARP and Cleaved-Caspase3 did not increase correspondingly after combination (data not shown). Excessive accumulation of ROS originates from damaged mitochondria and further activates the ROS-MAPK pathway. Furthermore, as demonstrated in Additional file 2: Fig. S2G, H, ROS was found to activate the JNK-MAPK pathway in NCI-H1975 cells. Mitochondrial membrane potential is broken and cytochrome C is released, which activates Caspase-9 and further activates Caspase-3 and Caspase-7, resulting in cell apoptosis [43]. Although autophagy can remove ROS or damaged mitochondria to a certain extent, FJHQ inhibits the protective autophagy of NSCLC cells and enhances the efficacy of chemotherapy drugs. This synergistic effect can be reversed by NAC, a ROS scavenger. FJHQ exhibits the same effects in vivo. Treatment of FJHQ with cisplatin daily for up to18 days exerted potent anti-tumor activity against NCI-H1299 cells xenografts with an excellent tolerance throughout the entire treatment process. These observations reflect the potential clinical importance of FJHQ for the treatment of NSCLC.

**Fig. 8:**
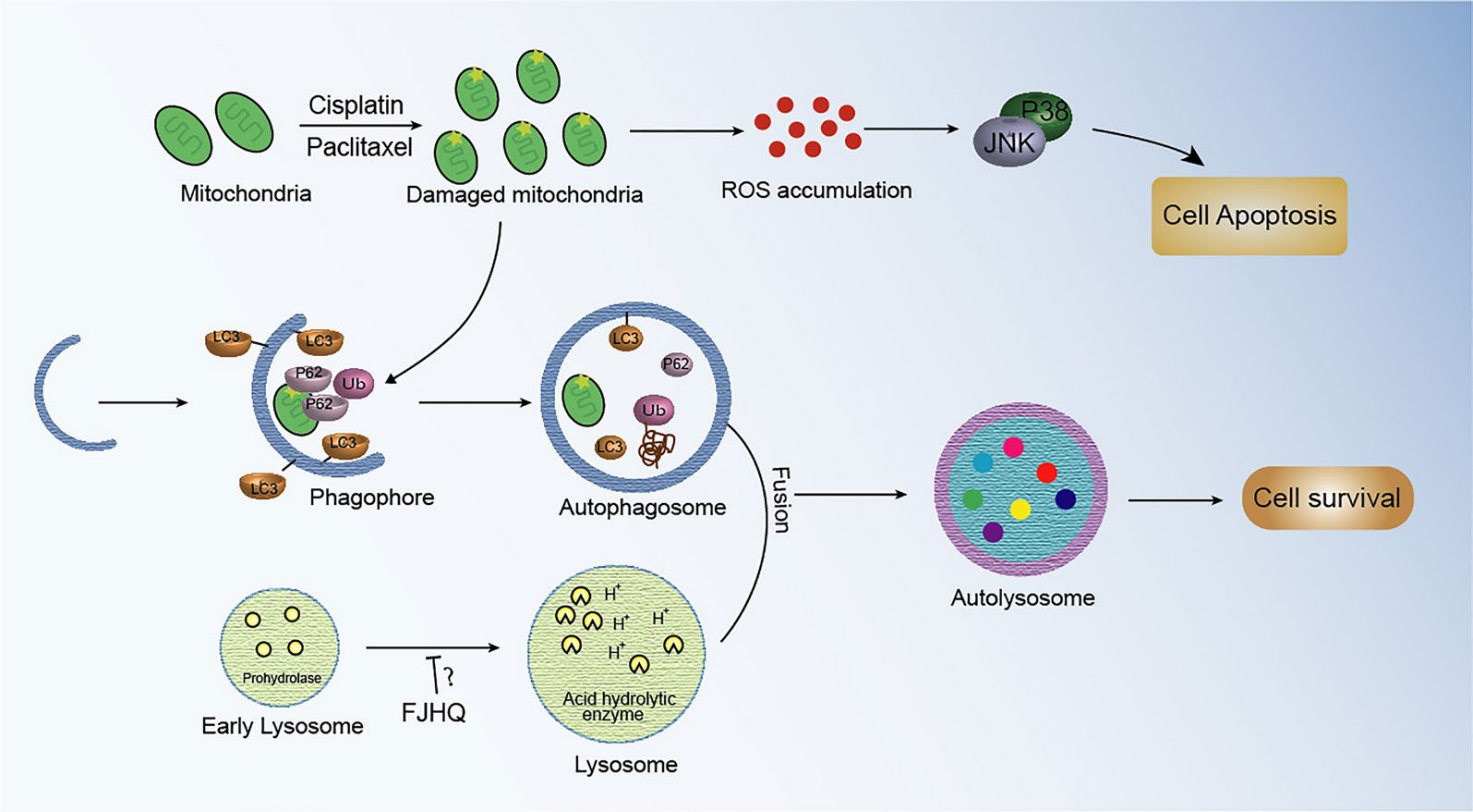
Mechanism of FJHQ inhibition of autophagy and synergistic effect of FJHQ and chemotherapy drugs. Cisplatin and paclitaxel induce mitochondrial damage to release ROS. However, the autophagy pathway removes damaged mitochondria and weakens the cytotoxic effects of chemotherapy drugs. As a novel late-stage autophagy inhibitor, FJHQ inhibits the maturity of cathepsin in lysosome, thus blocking autophagy pathway. When combined with FJHQ, the damaged mitochondria cannot be cleared through the normal autophagy pathway, leading to excessive ROS accumulation and further activation of the MAPK pathway. *FJHQ* Fangjihuangqi Decoction, *Cis* Cisplatin, *PTX* Paclitaxel:

In conclusion, we demonstrated for the first time that FJHQ decoction can interfere with autophagy pathways, which sensitizes the chemotherapeutic drug-induced death of human cancer cells. Figure 8 provides a schematic of the mechanisms and interactions. In the future, further clinical studies are expected to demonstrate that FJHQ can improve chemotherapy efficacy or reduce treatment-related side effects by reducing the dosage of anticancer drugs.

## Conclusions

Our study provides extensive evidence that FJHQ blocks the autophagy pathway by affecting the degradation of autophagy cargo, and further activates the ROS-MAPK pathway with ROS accumulation; thereby enhancing cisplatin- and paclitaxel-mediated anticancer effects.

## Supplementary Information

**Additional file 1: Figure S1.** Identification of main components in Fangjihuangqi decoction by UPLC-Q-TOF-MS/MS

**Additional file 2: Figure S2.** FJHQ inhibits the late stage of autophagic flux and enhanced the anti-cancer effects of cisplatin in NCI-H1975. (A) FJHQ results in increased P62 and LC3-I to LC3-II conversion in the form of a time and concentration gradient in NCI-H1975. (B) FJHQ affects mature-CatD in NCI-H1975. (C) Pretreatment with FJHQ (800 μg/mL) enhanced cis-mediated inhibition at all tested concentrations (25 and 50 μM) in NCI-H1975. (*P < 0.05; ***P < 0.001; multiple comparison ANOVA; Q > 1.15, synergistic effect). (D) The apoptosis rate induced by FJHQ combined with cis was significantly higher than that induced by cisplatin treating alone (** P <0.01; multiple comparison ANOVA; Q > 1.15, synergistic effect). (E) Compared with cisplatin (50μM) treating alone, Pre-treatment with FJHQ (800μg/ml) significantly increased the Cis-induced ROS production in NCI-H1975. (F)The apoptosis rate of FJHQ combined with cisplatin could be reversed by NAC (* P <0.05; ** P <0.01; ***P < 0.001, multiple comparison ANOVA and Q < 0.85 indicated an antagonistic effect). (G&H) The combination of cisplatin (50 μM) with FJHQ (800 μg/mL) significantly increased the levels of JNK phosphorylation and the increased P-JNK was reversed by NAC

## Data Availability

The datasets supporting the conclusions of this article are included within the article and its additional files.

## Abbreviations

FJHQ: Fangjihuangqi decoction
Cis: Cisplatin
PTX: Paclitaxel
TNBC: Triple-negative breast cancer
DMEM: Dulbecco’s modified eagle medium
CCK8: Cell counting kit-8
CQ: Chloroquine
HCQ: Hydroxychloroquine
HBSS: Hank’s balanced salt solution
AO: Acridine orange
BafA1: Bafilomycin A1
Cat D: Cathepsin D
PARP: Poly (ADP-ribose) polymerase
PI: Propidium iodide
ROS: Reactive oxygen species
NAC: N-Acetylcysteine
