# Supplementary material for: Identification of Fangjihuangqi Decoction as a late-stage autophagy inhibitor with an adjuvant anti-tumor effect against non-small cell lung cancer
Source: Chin Med. 2023 Jun 7;18:68. doi: 10.1186/s13020-023-00770-4 (PMC10246325; doi:10.1186/s13020-023-00770-4)
Supplement: Supplementary file 1 — Additional file 1: Figure S1. Identification of main components in Fangjihuangqi decoction by UPLC-Q-TOF-MS/MS [file 13020_2023_770_MOESM1_ESM.pdf]

| Number | tR/min | [M+H]+/ (m/z) | Formula | Ppm | Chemical compound | Secondary mass spectrometry fragment | Chemical structure |
|---|---|---|---|---|---|---|---|
| 1 | 2.37 | 314.13846 | $C_{18}H_{19}NO_4$ | -0.7 | Laurolitsine | 58.0709,107.0514,151.0761,269.1169, 283.0833,299.1149,298.1072,314.1409 | 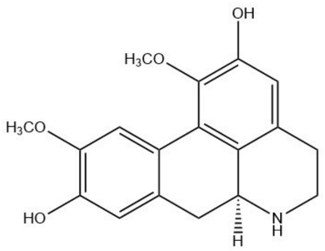 |
| 2 | 3.18 | 595.16575 | $C_{27}H_{30}O_{15}$ | -1.6 | Nicotiflorin | 134.0500,192.1060,271.1051,313.0671, 307.0617,321.0745,337.0689,355.0846, 379.0880,403.0765,421.0982,427.0971, 439.1092,457.1107,457.0887,481.0973, 475.0970,511.1251,541.1289,559.1499, 577.1477,595.2683 | 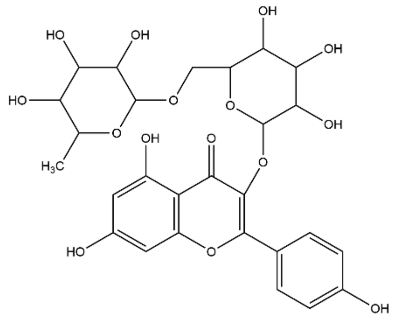 |
| 3 | 4.77 | 193.04891 | $C_{10}H_8O_4$ | -3.2 | Scopoletin | 56.9661,65.0432,75.0241,84.9566,92.8999, 103.0271,121.0286,133.9281,149.0820, 149.0208,150.0942,178.0855,178.0713, 193.0877,193.1057 | 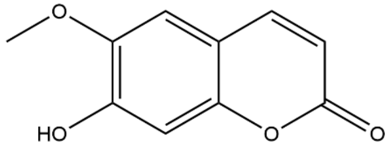 |
| 4 | 4.87 | 257.08084 | $C_{15}H_{12}O_4$ | -0.2 | Liquiritigenin | 81.0359,91.0560,119.0491,137.0225, 147.0426,211.0732,257.0784 | 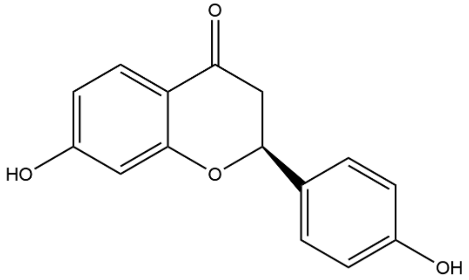 |

| | | | | | | | |
|---|---|---|---|---|---|---|---|
| 5 | 5.83 | 609.29542 | $C_{37}H_{40}N_2O_6$ | -0.7 | Fangchinoline | 367.1631,578.2507,609.2913 | 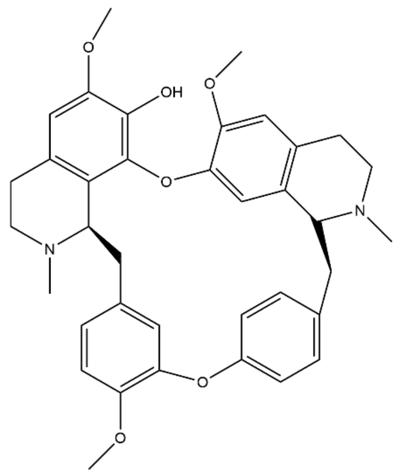 |
| 6 | 6.40 | 623.31112 | $C_{38}H_{42}N_2O_6$ | -0.7 | Tetrandrine | 623.308 | 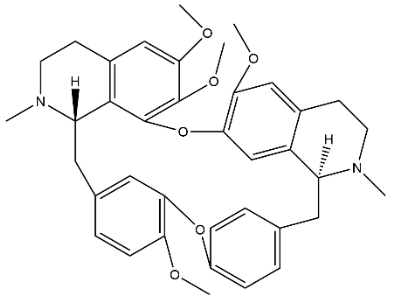 |
| 7 | 6.41 | 287.09128 | $C_{16}H_{14}O_5$ | -0.1 | LicochalconeB | 65.0400,84.9648,113.0586,123.0406,138.0282,147.0397,153.0561,167.0344,170.0962,181.0633,204.8821,204.9010,287.0903,287.1192 | 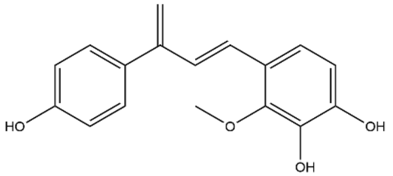 |
| 8 | 7.48 | 269.0808 | $C_{16}H_{12}O_4$ | -0.1 | Formononetin | 118.0416,170.0725,181.0641,197.0604,213.0901,226.0617,237.0533,253.0508,269.0809 | 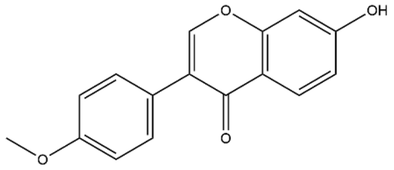 |

| | | | | | | | |
|---|---|---|---|---|---|---|---|
| 9 | 7.49 | 431.13366 | $C_{22}H_{22}O_9$ | 0.3 | Ononin | 269.079 | 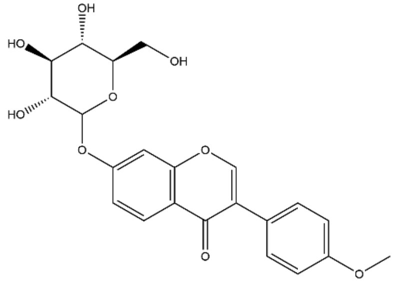 |
| 10 | 9.24 | 823.4104 | $C_{42}H_{62}O_{16}$ | -0.8 | Glycyrrhizic acid | 454.3450,453.3339,471.3460,647.3761 | 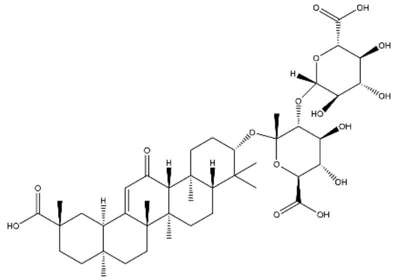 |
| 11 | 9.25 | 471.34659 | $C_{30}H_{46}O_4$ | -0.6 | 18β-Glycyrrhetinic Acid | 135.1192,173.1333,189.1631,235.1735, 317.2198,441.3193,453.3266 | 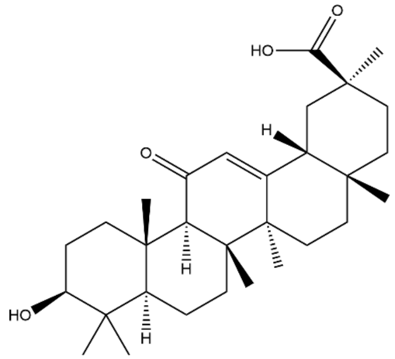 |
| 12 | 9.98 | 231.13742 | $C_{15}H_{18}O_2$ | -2.3 | Atractylenolide I | 77.0418,141.0012,157.1035,158.0265, 214.0888,231.1376 | 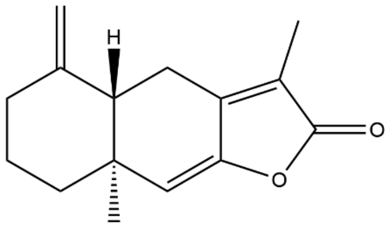 |

| | | | | | | | |
|---|---|---|---|---|---|---|---|
| 13 | 9.99 | 249.1481 | $C_{15}H_{20}O_3$ | -1.6 | Atractylenolide III | 55.9382,67.0588,69.0740,77.0429,79.0581, 83.0887,91.0569,95.0876,105.0713,119.0857, 128.0615,135.0451,149.0588,157.1032,161.0588, 163.0751,175.0755,189.0886,195.1209,203.1454, 213.1257,231.1364,231.1655 | 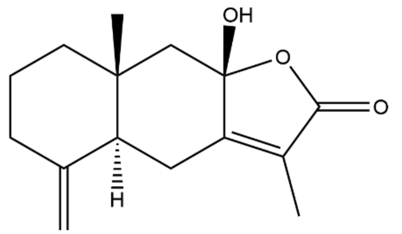  |
| 14 | 12.49 | 399.2166 | $C_{24}H_{30}O_5$ | -3 | Gancaonin T | 84.9687,122.9770,127.9993,141.9836,143.9942, 155.9973,158.0100,170.0082,172.0253,184.0262, 186.0462,214.0817,228.0954,258.8092,270.1388, 270.7608,294.2094,295.2148,312.8080,314.2000, 340.2157,358.2033,357.2024,399.3479 | 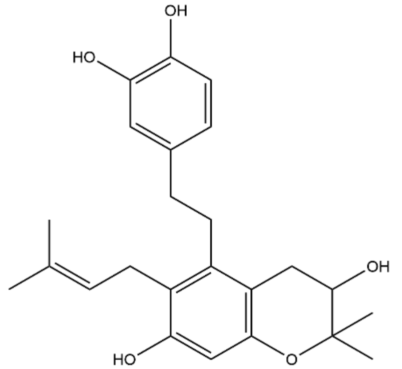  |
| 15 | 12.73 | 383.22169 | $C_{24}H_{30}O_4$ | -0.4 | Gancaonin R | 101.9517,314.1836,318.2111,320.2264,337.2006, 337.2144,383.2204 | 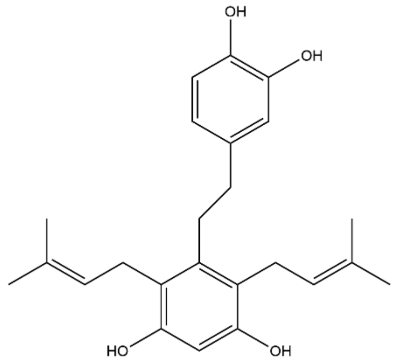  |

**Figure S1. The main components' detailed information of Fangjihuangqi decoction**
