# Supplementary figures and images for: Identification of Fangjihuangqi Decoction as a late-stage autophagy inhibitor with an adjuvant anti-tumor effect against non-small cell lung cancer
Source: Chin Med. 2023 Jun 7;18:68. doi: 10.1186/s13020-023-00770-4 (PMC10246325; doi:10.1186/s13020-023-00770-4)

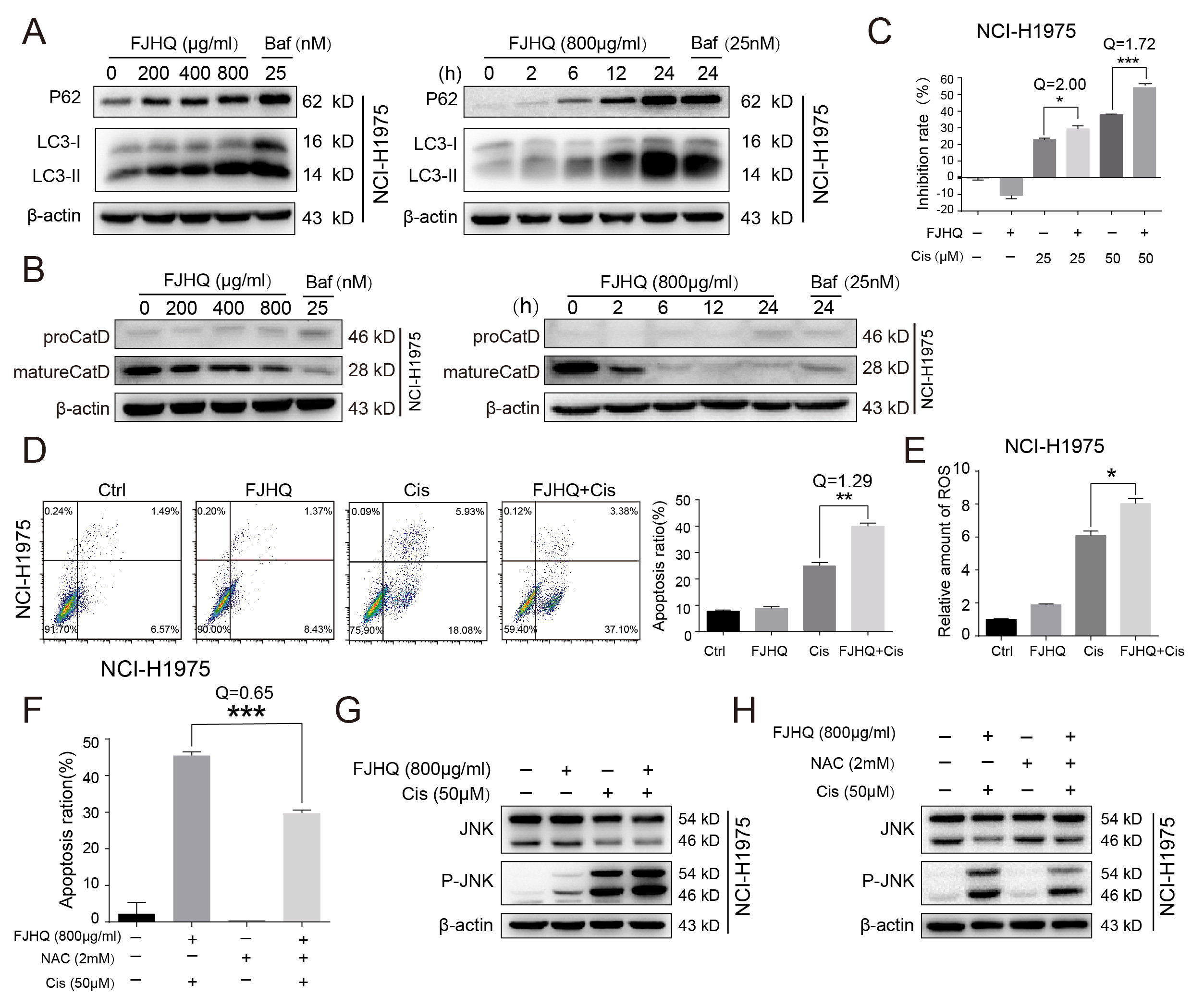

Supplement: Supplementary file 2 — Additional file 2: Figure S2. FJHQ inhibits the late stage of autophagic flux and enhanced the anti-cancer effects of cisplatin in NCI-H1975. (A) FJHQ results in increased P62 and LC3-I to LC3-II conversion in the form of a time and concentration gradient in NCI-H1975. (B) FJHQ affects mature-CatD in NCI-H1975. (C) Pretreatment with FJHQ (800 μg/mL) enhanced cis-mediated inhibition at all tested concentrations (25 and 50 μM) in NCI-H1975. (*P < 0.05; ***P < 0.001; multiple comparison ANOVA; Q > 1.15, synergistic effect). (D) The apoptosis rate induced by FJHQ combined with cis was significantly higher than that induced by cisplatin treating alone (** P <0.01; multiple comparison ANOVA; Q > 1.15, synergistic effect). (E) Compared with cisplatin (50μM) treating alone, Pre-treatment with FJHQ (800μg/ml) significantly increased the Cis-induced ROS production in NCI-H1975. (F)The apoptosis rate of FJHQ combined with cisplatin could be reversed by NAC (* P <0.05; ** P <0.01; ***P < 0.001, multiple comparison ANOVA and Q < 0.85 indicated an antagonistic effect). (G&H) The combination of cisplatin (50 μM) with FJHQ (800 μg/mL) significantly increased the levels of JNK phosphorylation and the increased P-JNK was reversed by NAC [file 13020_2023_770_MOESM2_ESM.jpg]
